# Antibacterial and Antibiofilm Properties of Self-Assembled Dipeptide Nanotubes

**DOI:** 10.3390/ijms24010328

**Published:** 2022-12-25

**Authors:** Iris Soares, Inês Rodrigues, Paulo Martins da Costa, Luís Gales

**Affiliations:** 1 i3S—Instituto de Investigação e Inovação em Saúde, Rua Alfredo Allen, 208, 4200-135 Porto, Portugal; 2 IBMC—Instituto de Biologia Molecular e Celular, Universidade do Porto, Rua Alfredo Allen, 208, 4200-135 Porto, Portugal; 3 CIIMAR—Interdisciplinary Centre of Marine and Environmental Research, University of Porto, Avenida General Norton de Matos, 4450-208 Matosinhos, Portugal; 4 ICBAS—Instituto de Ciências Biomédicas Abel Salazar, Rua de Jorge Viterbo Ferreira, 228, 4050-313 Porto, Portugal

**Keywords:** dipeptides, supramolecular assemblies, nanotubes, antibacterial activity, antibiofilm activity, diphenylalanine

## Abstract

Over recent decades, multidrug-resistant pathogens have become a global concern, with WHO even considering it one of the biggest threats to global health, food security, and development today, which led to the search for alternative antibacterial agents. A special class is formed by peptides composed by the diphenylalanine motif whose antibacterial properties result from their supramolecular arrangement into nanotubes. However, several other dipeptides that also form nanotubes have been largely overlooked. Here, we present the antibacterial activity of four dipeptide nanotubes. The results point to diverse mechanisms through which dipeptide nanotubes exert their effect against bacteria. Antibacterial activity was similar for dipeptide nanotubes sufficiently wide to allow water flux while dipeptides displaying smaller channels were inactive. This suggests that two of the tested dipeptides, L-Phe-L-Phe (FF, diphenylalanine) and L-Leu-L-Ser (LS), are pore forming structures able to induce membrane permeation and affect cellular hydration and integrity. Of these two dipeptides, only FF demonstrated potential to inhibit biofilm formation. The amyloid-like nature and hydrophobicity of diphenylalanine assemblies are probably responsible for their adhesion to cell surfaces preventing biofilm formation and bacteria attachment.

## 1. Introduction

A major health problem is contamination by bacteria, such as *Salmonella* spp., *Shigella* spp., *Micrococcus* spp., Enterococcus faecalis, Bacillus licheniformis, Escherichia coli, Listeria monocytogenes, Staphylococcus aureus, Campylobacter jejuni, Yersinia enterocolitica, Vibrio parahemolyticus, Escherichia coli 0157:H7, and Clostridium botulinum [1]. Different mechanisms have been used to mitigate such contaminations, such as addition of sachets/pads containing volatile antimicrobial agents into packages, incorporation of volatile and non-volatile antimicrobial agents directly into polymers, coating or adsorbing antimicrobials onto polymer surfaces, immobilization of antimicrobials to polymers by ion or covalent linkages, and use of polymers that are inherently antimicrobial, such as antimicrobial peptides (AMPs) [2].

AMPs are known to have broad spectrum activity and to cause less bacterial resistance than regular antibiotics; however resistance mechanisms can still be prompted, usually by alteration of membrane composition, peptidase expression, or peptide-efflux pumps [3]. The formation of the extracellular biofilm matrix may contribute significantly to bacterial resistance by electrostatic repulsion and/or sequestration of antibacterial substances [4,5]. AMPs have usually a cationic nature, which enables the interaction between the AMP and the negatively charged bacterial cell membrane or wall. Furthermore, AMPs tend to be amphiphilic, and are capable of folding into α-helical or β-sheet motifs when in contact with the membrane, leading to its disruption or to translocation and interaction with cell components. Despite the increasing interest in AMPs, most studies thus far have focused on naturally occurring, but complex peptides and protein [6].

An emergent approach is the fabrication of antibacterial nanostructures through self-assembly of peptides [7,8]. These new systems achieve function by a holistic approach that considers sequence, self-assembly and interaction over-time, while keeping the minimalist approach in the design of the sequences, preferring short peptides over hole proteins. In some cases, proteins aggregate to form unwanted structures, which can have dangerous consequences such as in the case of the amyloid β-peptide involved in the Alzheimer’s disease [9,10]. On the other hand, aggregation can be leveraged for new fluorescent properties in peptide probes. There have been promising results of peptides with J-aggregates, as well as with carbon nanotubes as means of biological applications in probing and biosensing [11,12,13,14].

Dipeptides have been used to produce microporous solids, homogeneous [15] or hybrid by coordination of metal ions [16,17,18]. Hydrophobic dipeptides in particular were found to crystallize into helical arrangements leading to the formation of nanochannels [19] that are able to uptake small guest molecules. For instance, they can distinguish between gas molecules with similar size, a feature relevant in many chemical engineering processes [20,21,22,23,24,25]. Peptides may also be used as vehicles for controlled delivery of gas molecules in biomedical applications, such as of nitric oxide for wound healing [26] or fluorinated ethers in use as anesthetics [27].

From all dipeptides, diphenylalanine (FF) is the most prominently studied resulting in a broad range of potential nanotechnological applications [28]. The FF motif is extremely versatile, self-assembling into distinct nanomorphologies such as nanotubes, nanowires, nanovesicles, nanotapes, nanocrystals, necklaces and nanofibers by varying experimental conditions. Some of these structures display interesting properties such as photoinduced ferroelectricity and piezoelectricity setting for functional nanomaterials that respond to electrical or mechanical stimuli [29]. Crucially, Gazit and colleagues reported the antimicrobial activity of FF nanostructures [6], and showed that supramolecular FF induces substantial disruption to Gram-negative bacterial morphology and causes membrane permeation. Inspired by this finding, we will investigate the antimicrobial properties FF together with three other dipeptides, that also self-assembled into tubular structures, Leucyl-Serine (LS), Isoleucine-Valine (IV) and valine-Isoleucine (VI) [30,31]. Furthermore, the incorporation of antimicrobial agent iodine in the crystals, previously reported for LS [31] was replicated for all dipeptide crystals, in order to ascertain the potential of iodine as facilitator of the antimicrobial activity.

## 2. Results

### 2.1. Dipeptide Nanotubes

**Figure 1:**
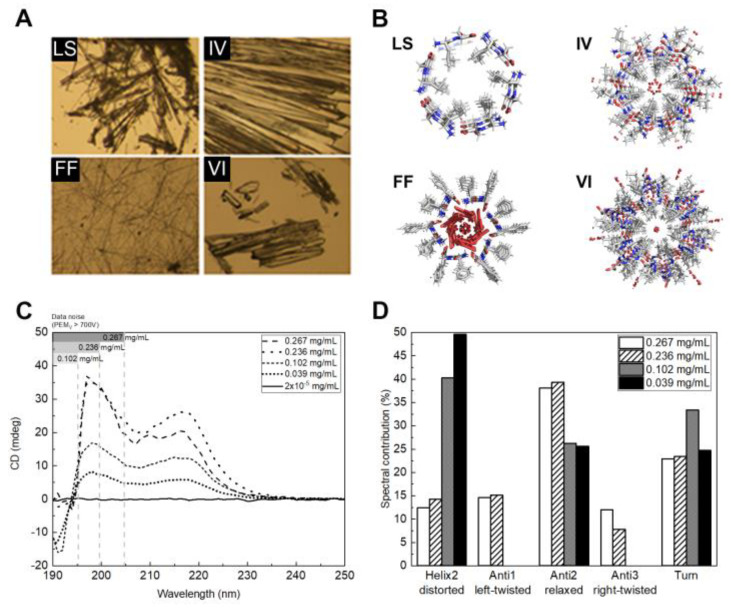
(**A**) Optical microscope image of LS, FF, IV and VI crystals; (**B**) Crystal structures of the dipeptides nanotubes along the c crystallographic axis; (**C**) CD spectra for FF crystals at different concentrations; and (**D**) spectral contribution of the different secondary structures for the several crystal concentration (mg/mL) sample.

The four dipeptides were crystallized to yield millimeter-sized needle-like crystals (Figure 1A) that were used to collect X-ray diffraction data with an in-house diffractometer. The crystal structures of LS and VI were similar to the ones we have previously obtained [21] and deposited with the Cambridge Crystallographic Data Centre (CCDC 749867-LS and 749868-VI). The crystal structures of FF and IV are similar to the ones obtained originally by Gorbitz [19,30].

Crystals structures reveal that the dipeptides assemble into helical networks of hydrogen-bonded main-chains around a central channel (Figure 1B). Dipeptides crystallize in their zwitterionic state and have been systematically characterized by Görbitz, that identified two main classes of hydrophobic dipeptides [32], the VA-class and the FF-class, named after the first dipeptide discovered in each class. IV and VI belong to the VA-class, with dipeptide side-chains pointing inwards to the center of the channels, forming hydrophobic pores with diameters of 3.9 Å (IV) and 3.7 Å (VI). In FF, six molecules form a ring with the side chains pointing outwards; water filled channels, with 10 Å diameter, are formed by translation of the rings. Interestingly, FF may also be self-assembled into nanotubes or hollow fibers containing much larger channels, in the order of several nm. The walls of this nanotubular structures present the same molecular packing of the X-ray quality diffracting crystals [33]. The mechanisms to produce the diverse types of FF assemblies are elusive but crystallization from pure water seem to yield mostly solid FF crystals. LS is an exception to the rule that only dipeptides with two hydrophobic side-chains form porous structures. It forms hydrophobic pores with 4.9 Å diameter decorated by the side chains of the leucine residues.

**Figure 2:**
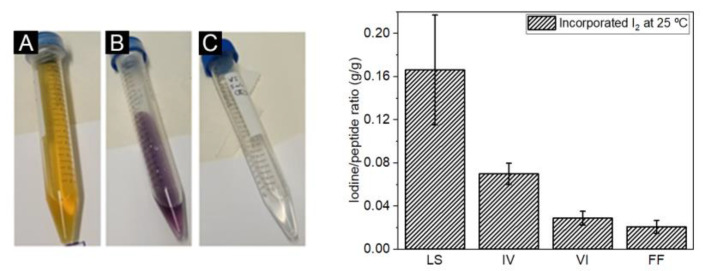
Left: peptide solution during iodine titration showing the solution after the addition of (**A**) potassium iodide, (**B**) soluble starch solution and (**C**) at the end of titration. Right: iodine content (in g of iodine/gram peptide) after I_2_ incubation for 24 h at 25 °C.

Solutions of FF nanostructures were also analyzed by Circular Dichroism (Figure 1C). Secondary structure percentages (Figure 1D) were determined using the BeStSel (Beta Structure Selection) web server (https://bestsel.elte.hu/index.php (accessed on 20 April 2022)) in the 190–250 nm range where dynode voltage was lower than 700 V) and structure was concluded to be 49.6% helix_2_ (distorted), 25.6% anti_2_ (relaxed) and 24.7% turns for a concentration of 0.039 mg/mL of FF and then, as FF concentration increases to 0.267 mg/mL, helix_2_ (distorted) decreases, to 12.4%, anti_2_ (relaxed) increases to 38.1, turns remain similar, anti_3_ (right-twisted) and anti1 (left twisted) begin to contribute to the structure, up to a contribution of 12% and 14.6%, respectively. The spectra remain identical after sample storage over one week. The solubility of FF in water was determined to be 0.76 g/L [34] however, as observed in Figure 2, pre-formed FF nanostructures do not dissolve into disordered single molecules, at least completely, after dilution to sub-critical concentrations.

### 2.2. Iodine Incorporation

Dipeptide crystals were impregnated with iodine and turned to a purple color, an indication of iodine uptake. Iodine content was determined by redox titration (color change illustrated in Figure 2).

Iodine content of dipeptide nanostructures presented some variability (Figure 2) presumably due to defects in the crystalline frameworks or due to pore blocking by incomplete removal of solvent molecules. LS is able to uptake more iodine than the other dipeptides which is not surprising as strong interactions are formed between this guest molecule and the LS channel surface, as revealed by Görbitz [31]. Iodine uptake is close to saturation as calculated by the crystal packing. IV and VI frameworks seem to be flexible enough to enable the incorporation of iodine up to some extent. VI display narrow channels which is consistent with lower adsorption of I_2_. FF crystals display channels with approximately 10 Å of diameter. Pore diffusion of I_2_ should be much faster in FF than in the other crystals. Possibly I_2_ guest molecules are lost within seconds prior to quantification.

### 2.3. Antibacterial Activity of Dipeptide Nanotubes

Dipeptides were evaluated for their activity against bacterial reference strains and multidrug-resistant isolates, and their Minimal Inhibitory Concentration (MIC) and Minimal Bactericidal Concentration (MBC) values are shown (when determined) in Table 1.

IV and VI showed no antimicrobial activity within the tested concentrations. FF and LS were used as crystalline nanoassemblies (Table 1) and after recrystallization towards macro-crystals, a process that in general did not improve the antibacterial activity. Dipeptides nanostructures were also tested after uptake of iodine. It is interesting to note that iodine did not significantly improve their antibacterial properties. Iodine’s microbicidal activity is known to involve the oxidation of bacterial cell components eventually producing the simultaneous inactivation of bacterial enzymes, a loss of genome integrity, and cell wall damage [35]. Limitations in the uptake of iodine due to the dipeptide crystals low porosity (down to 5%), combined with the inability to control the I_2_ release kinetics, which should be very fast in FF and very slow in LS, are probably responsible for the marginal gain in antibacterial efficacy.

MIC values for LS and FF were similar; for *E. faecalis* ATCC 29212, between 0.4 mg/mL and 0.75 mg/ mL, and 1.5 mg/mL for *E. coli* ATCC 25922 and *S. aureus* ATCC 29213. Furthermore, even when MIC values were above the highest tested dipeptide concentration, the number of bacteria observed lowered as the dipeptide concentration increased. This effect was specially noted in the case of *E. coli* ATCC 25922, indicating that MIC value might be close to the maximum concentration tested. According to [36], iodinated LS is bactericidal for *E. coli* ATCC 25922, as well as iodinated FF for *E. faecalis* ATCC 29212 and FF and LS for *S. aureus* ATCC 29213.

### 2.4. Synergistic Association with Antibiotics

FF and LS synergistic association (checkerboard assay) with antibiotics to which reference strains are “intrinsically” resistant [37] and the multidrug-resistant strains have developed an “acquired” resistance mechanism [38]. Regarding reference strains, only the combination effect of FF and LS with kanamycin (KAN) against *E. facealis* ATCC 29212 showed a clear synergistic effect (ΣFIC < 0.5, Table 2). The remaining combinations showed a FICI between 0.5 and 4, indicating a “no interaction” effect. The combination effect of FF and LS with oxacillin, vancomycin and cefotaxime against MRSA, VRE and ESBL isolates, respectively, was found to be indifferent (ΣFIC > 0.5). These results indicated that, although FF and LS are not strong antimicrobial by themselves, when conjugated with KAN increase the effect of this drug on *E. faecalis* ATCC 29212.

### 2.5. Effect in Membrane Permeability

*E. coli* and *S. aureus* were selected to continue FF and LS evaluation because of their clinical relevance to human medicine. Membrane permeability assays were performed using 8-Anilinonaphthalene-1-sulfonic acid (ANS) as a fluorescence probe. ANS is a hydrophobic probe that fluoresces weakly in aqueous environments but exhibits enhanced fluorescence in nonpolar/hydrophobic environments and has been widely used to monitor changes in membrane permeability. ANS is size excluded from the channels formed by LS and FF so a hypothetic increase in ANS membrane permeation should arise from a disruption of the membrane structure.

**Figure 3:**
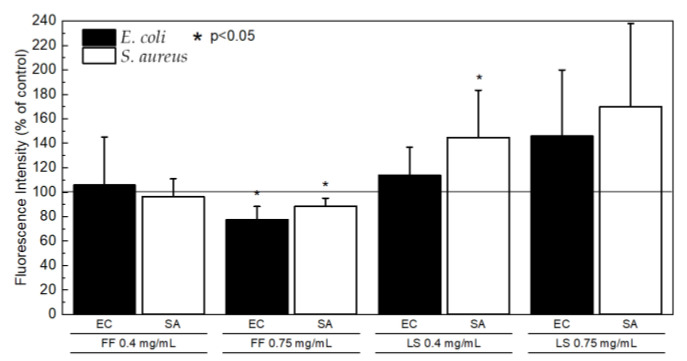
Membrane permeability for *E. coli* ATCC 25922 (black) and *S. aureus* ATCC 29213 (white) shown as percentage of control.

LS seems to slightly increase the permeability to the fluorescent probe while FF apparently produces the opposite effect (Figure 3).

### 2.6. Inhibition of Biofilm Formation

**Figure 4:**
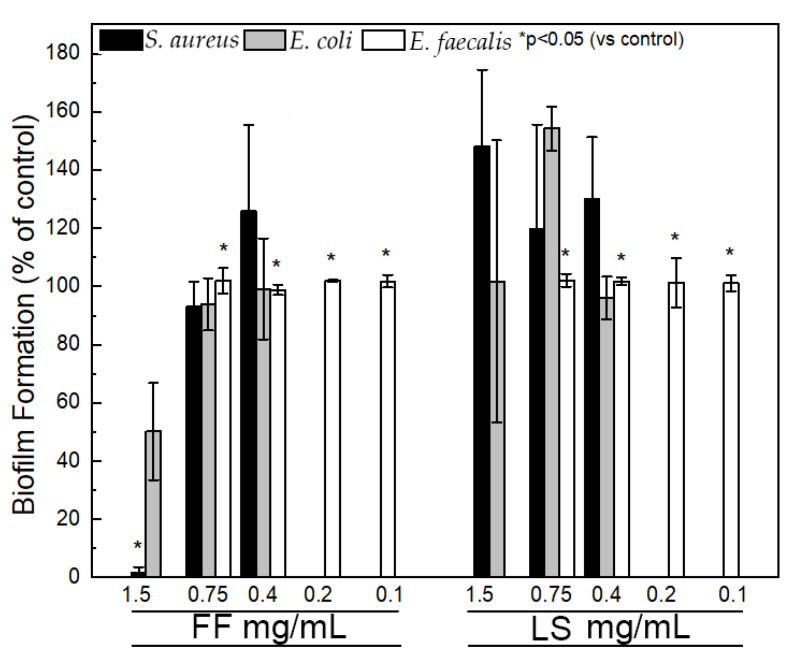
Biofilm formation for *S. aureus* ATCC 29213 (black), *E. coli* ATCC 25922 (gray) and *E. faecalis* ATCC 29212 (white), shown as percentage of control.

Assays were performed against *E. faecalis* ATCC 29212 (lowest MIC) and *E. coli* ATCC 25922 and *S. aureus* ATCC 29213, selected because of their clinical relevance. The effect of FF and LS at different concentrations (ranging from 0.1 to 1.5 mg/mL) on biofilm formation was assessed by biomass quantification through the crystal violet assay (results shown in Figure 4). Above the critical concentration, FF (1.5 mg/mL) exhibits significant inhibition of biofilm formation for *S. aureus* (98.5 ± 2.5%), moderate inhibition of *E. coli* (49.8 ± 16.7%). For FF concentrations up to 0.75 mg/mL there was no inhibition of biofilm formation, even for *E. faecalis.* LS did not inhibit biofilm formation at all. In fact, the polarity of the serine residue seems to increase the affinity of the dipeptide structures towards the extracellular matrix and increase slightly biofilm formation.

### 2.7. Effect on Cell Viability

**Figure 5:**
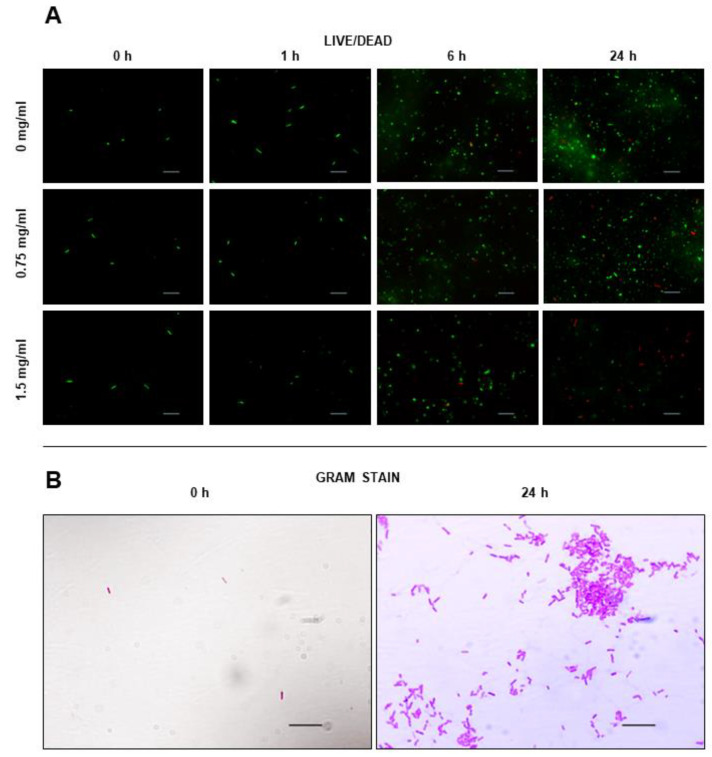
(**A**) Live/Dead staining of *E. coli* ATCC 25922 cells with an FF concentration of 0 mg/mL, 0.75 mg/mL and 1.5 mg/mL at 4 timepoints (0 h, 1 h, 6 h and 24 h); (**B**) Gram staining of *E. coli* ATCC 25922 at 2 timepoints (0 h and 24 h). Scale bar in (**A**,**B**): 10 µm.

The effect of FF nanostructures on cell viability was evaluated using the LIVE/Dead assay (Figure 5). In this assay, cells are colored with two probes, with the green fluorescence of Syto9 probe indicates alive cells, due to its ability to penetrate bacterial cells and stain nucleic acid, while red fluorescence of Propidium Iodide (PI) is not permeant to live cells, being used for detection of dead cells.

The addition of FF seemed to impair bacterial growth, being this effect more noticeable for FF concentration of 1.5 mg/mL. Cell number decreased only slightly for FF concentration of 0.75 mg/mL. The threshold around 0.75 mg/mL suggests that FF effect arises from the supramolecular assemblies and not from the single molecules (FF solubility 0.76 g/L), although we have checked by CD that at least part of the FF assemblies persist after dilution down to sub-critical concentrations.

## 3. Discussion

Antibacterial properties of four dipeptides that form supramolecular nanotubular structures were investigated. The results suggest diverse mechanisms through which dipeptides simultaneously exert their effects.

Dipeptide assemblies, at least in the concentrations used in this work, do not seem to promote bacterial membrane disruption and improve permeation to large molecules, such as antibiotics. As synergism was only observed with one of the antibiotics evaluated, it hardly arises from a general mechanism of membrane disruption induced by the dipeptide nanostructures. It was already shown by Silva and colleagues that drugs can be successfully conjugated with diphenylalanine nanotubes and homogeneously embedded in the dipeptide structure [39]. We did not perform the characterization of the mixtures of dipeptide assemblies with drugs but presumably conjugation of kanamycin is attained.

It was observed by Laverty and colleagues [40] using scan electron microscopy that FF was able to disrupt the peptidoglycan cell wall and cytoplasmic lipid membrane of Gram-positive *S. aureus* in a concentration dependent manner, but with evident effects only observed with FF concentrations significantly higher (2.5–5 mg/mL) than the ones tested here. Others have also observed that FF increases the permeability of the outer membrane of Gram-negative bacteria to fluorescent probes [6,40], a trend that we did not observe (Figure 3).

However, dipeptides may increase membrane permeation to water and affect cellular hydration and integrity. In fact, the efficacy seems to depend on the size of the channels formed by the dipeptide tubular structures. Peptide assemblies with channel diameters >4 Å (FF and LS) show similar antibacterial activity while the ones with narrow channels (<4 Å, IV and VI) are inactive. Zhao et al. showed by atomistic simulation that IV and VI allow only relatively restricted transport of water molecules, much smaller than LS water flux [41]. FF was not investigated by the authors, but the crystal structure of this dipeptide shows that it assembles in water filled nanotubes [19]. LS possess high salt rejection (Na^+^ and Cl^−^) [41] which suggests that antimicrobial activity arises from a sudden increase in the membrane permeability to water. Thus, our results seem to be in agreement with the findings of Gazit and colleagues that dipeptide nanostructures may form pores in inner and outer bacterial membranes [6].

In general, biofilm forms are associated with increased tolerance to antibiotics, requiring 10–1000 times antibacterial concentrations to achieve equivalent planktonic efficacy [42]. It is conceivable that the large supramolecular structures investigated in this work are size excluded from the biofilm matrix produced by bacteria, prolonging bacterial survival to higher dipeptide concentrations. We observed that inhibition of bacterial biofilm formation seems to be a specificity of FF assemblies and is only observed above FF critical concentration. Eradication of mature biofilm forms by FF assemblies at concentration around 5–10 mg/mL was already noticed by Laverty and colleagues [40]. FF assemblies are amyloid-like structures, displaying an hydrophobic surface decorated by aromatic groups and a propensity to adhere to inanimate surfaces and host cells [43]. These nanostructures may readily adsorb to the cell surfaces preventing bacteria attachment. FF entrapment of cells may also interfere with cell division and bacterial growth (Figure 5). A similar mechanism of protection by the amyloid-beta peptide against microbial infection in mouse and worm models of Alzheimer’s disease was already proposed [44].

## 4. Materials and Methods

### 4.1. Crystal Growth of Nanotubular Dipeptides

FF, LS, VI and IV peptides were purchased from Bachem (Bubendorf, Switzerland). To obtain FF crystals, the dipeptide was dissolved in ultrapure water at a concentration of 3 mg/mL at 80 °C and kept at that temperature for 30 min before being slowly cooled to room temperature. LS crystals were grown through phase inversion of an aqueous solution by acetonitrile as previously described [21]. Briefly, the sitting drop vapor diffusion method was applied, with 5 µL drops of LS dissolved in ultrapure water at a concentration of 230 mg/mL equilibrated with 200 µL of acetonitrile on each well. IV crystals and VI crystals were obtained by solvent evaporation at 60 °C for 24 h, from 7.4 mg/mL solutions of the dipeptides in ultrapure water.

### 4.2. Single Crystal X-ray Diffraction

Single crystals of each of the dipeptides were mounted on a cryoloop using paratone. X-ray diffraction data were collected at room temperature with a Gemini PX Ultra equipped with CuKα radiation (λ = 1.54184 Å) (Rigaku (Austin, TX 78717 USA) /Oxford Diffraction). The structures were solved by direct methods using SHELXS-97 and refined with SHELXL-97 [45].

### 4.3. Circular Dichroism

Samples of pre-formed FF crystals that were diluted in 9 sequential 2-fold dilutions in ultrapure water, were analyzed by Circular Dichroism, using a JASCO 815 instrument, fitted with a Peltier temperature controller fixed at 25 °C, using a quartz cuvette with an optical path of 0.1 mm (Hellma Analytics, Germany). Data acquisition was performed in 1 nm increments with a spectral bandwidth of 1 nm and a 2 s integration time, with an acquisition speed of 50 nm/min. The spectrum of each sample was taken trice and averaged. Spectra were corrected with ultrapure water spectrum, acquired in the same conditions. FF concentration was determined for each sample using a Thermo Scientific NanoDrop^TM^ 1000 Spectrophotometer (Thermo Fisher Scientific, Waltham, MA, USA), by measuring absorbance at 256 nm, with a pathlength of 1 mm for 2 µL samples, using ultrapure water as blank sample. A calibration curve was previously obtained for sample quantification, using the same method to obtain spectra of 9 FF solutions, at concentrations of 0.67 mg/mL, 0.60 mg/mL, 0.50 mg/mL, 0.47 mg/mL, 0.40 mg/mL, 0.34 mg/mL, 0.27 mg/mL, 0.20 mg/mL, 0.13 mg/mL, 0.067 mg/mL and 0 mg/mL.

### 4.4. Iodine Incorporation

All crystals were incubated in I_2_ by placing a vial with the dry crystals in glass media bottle sealed with PTFE tape that contained I_2_ powder (Merk, Germany, resublimed). The crystals were kept in the vial, at room temperature for one week.

Iodine content was accessed by titration. For this, the samples were washed with 2 mL KI solution at a concentration of 10% (Merk, Germany) and then titrated with sodium thiosulfate 1 mM (Merk, Germany) until the solution became clear. Soluble starch solution at a concentration of 20% (Merk, Germany) was then added, turning the solution purple, and the solution was again titrated until clear with sodium thiosulfate solution.

Iodine content was calculated by

(1) $$\frac{cNa_{2}O_{3}S_{2}\cdot 5H_{2}O\times vNa_{2}O_{3}S_{2}\cdot 5H_{2}O\times 253.8\times 10^{-3}}{2}$$

where $cNa_{2}O_{3}S_{2}$ and $vNa_{2}O_{3}S_{2\;}$are the concentration and volume of sodium thiocyanate used for the titration of each sample, $253.8\times 10^{-3}$ is the molar mass of I_2_ in mol/mg and 2 is the number of electrons exchanged in the redox reaction. Crystals with incorporated I_2_ are from here on referred as FF·I_2_, LS·I_2_, VI·I_2_ and IV·I_2_.

### 4.5. Bacterial Strains

Seven strains were used to investigate the antimicrobial effect of dipeptides. Four reference strains, two Gram-negative *(Escherichia coli* ATCC 25922, *Pseudomonas aeruginosa* ATCC 27853) and two Gram-positive (*Staphylococcus aureus* ATCC 29213, *Enterococcus faecalis* ATCC 29212), purchased from American Type Culture Collection (ATCC) were used, as well as three multi-drug resistant isolates, encompassing an Extended-Spectrum β-Lactamase (ESBL)-producing *E. coli* (ESBL-SA/2), a Methicillin-Resistant *S. aureus* (MRSA-66/1) [46], and a Vancomycin-Resistant *E. faecalis* (VRE-B3/101) [47]. All bacterial isolates were stored at −80°C until use and, before each assay, plated in Mueller Hinton (MH) agar (Biokar Diagnostics, Allone, France) for 18 to 24 h at 37 °C.

### 4.6. Antibacterial Assays

Determination of Minimal Inhibitory Concentration (MIC) was conducted by broth microdilution method, following the Clinical and Laboratory Standards Institute (CLSI) guidelines [37], with minor modifications. In short, bacteria were inoculated in 0.1× Tryptic Soy Broth (TSB) (Biokar Diagnostics) to achieve a final concentration of $5\times 10^{5}$ CFU/mL in each microplate well. A volume of 50 µL of the test dipeptide was serial diluted at different concentrations in a 96 well plate, ranging from 1.5 mg/mL to 5 µg/mL, along with 50 µL of bacterial inoculum. After 20 h incubation at 37 °C under aerobic conditions, the MIC was determined, being established as the lowest compound concentration at which no visible bacterial growth occurred. The Minimal Bactericidal Concentration (MBC) was determined by subculturing 10 μL from each well without apparent microbial growth on MH agar and then they were incubated overnight at 37 °C. MBC was considered as the lowest compound concentration that kills 99.9% of the initial bacterial population. By the formal definitions, a bactericidal antibiotic is one for which the MBC/MIC ratio is ≤4, while a bacteriostatic agent has an MBC to MIC ratio of >4 [36]. These assays were done in triplicate for the four reference strains. As a positive control, 100 µL of inoculum was used and, as a negative control, compound and medium without added bacterial inoculum were also performed. One negative control per plate and one positive control per bacteria were used.

### 4.7. Checkerboard Assay

Checkerboard assays were conducted to evaluate potential synergism between the supramolecular dipeptides and several clinically relevant antibiotics. Briefly, in a 96 well plate, dipeptides were serially diluted (double serial dilutions) along the ordinate, while the antibiotic was equally diluted along the abscissa. The final inoculum in each well was $5\times 10^{5}\;\mathrm{CFU}/\mathrm{mL}$ and then the plates were incubated for 24 h at 37 °C under aerobic conditions. Both MIC and MBC values were determined as previously described. The selection of antibiotics was based on the innate resistance (for reference strains) and on acquired resistance (for multi-drug resistant isolates): *E. coli* ATCC 25922 was tested with vancomycin (VAN) and erythromycin (ERI), *S. aureus* ATCC 29213 with ceftazidime (CAZ) and nalidixic acid (NA), *E. faecalis* ATCC 29212 with kanamycin (KAN) and cefotaxime (CTX), *P. aeruginosa* ATCC 27853 with ampicillin (AMP) and KAN, *E. faecalis* VRE-B3/101 with VAN, *E. coli* ESBL-SA/2 with CTX and *S. aureus* MRSA-66/1 with oxacillin (OXA). The fractional inhibitory concentration (FIC) was calculated as follows: FIC of crystal (FIC A) = MIC of crystal in combination/MIC of crystal alone, and FIC of antibiotic (FIC B) = MIC of antibiotic in combination/MIC of antibiotic alone. The sum of each FIC, The FIC index (ΣFIC), was interpreted as follows: FICI ≤ 0.5, ‘synergy’; 0.5 < FICI ≤ 4, ‘no interaction’; 4 < FICI, ‘antagonism’ [48]. The lowest ΣFIC was used to define synergy.

### 4.8. Membrane Permeability Assay

8-Anilinonaphthalene-1-sulfonic acid (ANS) uptake assay was performed in *E. coli* ATCC 25922 and *S. aureus* ATCC 29213. ANS has been broadly used to study biological systems due to its environmentally sensitive fluorescent nature and propensity to bind to hydrophobic surfaces [49]. Briefly, the bacteria were suspended in 10 mM Tris HCl, 150 mM NaCl buffer (pH 7.4) to an OD of 0.1 and added to a 40 µM ANS in 10 mM Tris HCl, 150 mM NaCl buffer (pH 7.4) in a ratio of 1:1 *v*/*v*, and then placed in a cool dark place for 20 min. The bacteria with ANS were then added to the dipeptides, in a black 96 well plate to a final compound concentration of 0.75 and 0.4 mg/mL. Fluorescence was measured at 460/40 after an excitation at 360/40. The 1% TritonX-100 and 10 mM Tris HCl, 150 mM NaCl buffer (pH 7.4 were used as control).

### 4.9. Biofilm Formation Inhibition Assay

*S. aureus* ATCC 29213, *E. coli* ATCC 25922 and *E. faecalis* ATCC 29212, as representative of the two major classes of bacteria (Gram-positive and Gram-negative), were selected to evaluate the antibiofilm activity of the dipeptides. Biofilm formation inhibition assay was conducted through quantification of total biofilm biomass, using the crystal violet method, as previously described [50,51]. For these essays, a bacterial suspension was dispensed into a 96 flat well plates at a final concentration of 1 × 10^6^ CFU/mL. In each well, 100 µL of bacterial inoculum and 100 µL of compound were added. FF and LS were tested at concentrations ranging from 1.5 mg/mL to 0.1 mg/mL. As a positive control, 200 µL of the inoculum were added to the well, and, as a negative control, 200 µL of TSB were added. Sterile ultrapure water was added to the wells of the edge of the plate to minimize edge effect. After incubation at 37 °C for 24 h, the planktonic phase of all samples was removed, and the samples were washed trice with 200 µL of PBS. Afterwards, the plates were dried at 60 °C for 1 h to promote biofilm adhesion to the plate reducing variability in the coloring stage. The plates were then removed from the heat, and 150 µL of 0.5% crystal violet added to all samples. After 5 min, the crystal violet was removed and the samples washed trice with 200 µL of PBS, and then dried at room temperature. Finally, acetic acid 33% was added to the samples and the absorbance measured at 570 nm using a Mark Microplate Absorbance Reader, Bio-Rad/Thermofisher Scientific Multiskan^®^ EX. Data are presented as percentage of control. Three independent assays were performed. All samples were performed in triplicate and three independent assays were performed.

### 4.10. Live Dead Assay

*E. coli* ATCC 25922 in the presence of FF was selected for the microscopic observation of cell viability through live dead assay. Briefly, 1 mL of bacterial inoculum (at final concentration of $5\times 10^{5}$ CFU/mL) with 1 mL of FF solution at 1.5 mg/mL or at 0.75 mg/mL were incubated at 37 °C for 0 h, 1 h, 6 h, and 24 h. Then, 1 mL of inoculum diluted in 1 mL of TSB medium was used as a positive control. After established time-points, 1 mL of the suspension was transferred and centrifuged at 10,000× *g* rpm for 10 min at room temperature. The supernatant was removed, and the pellet resuspended in 1 mL PBS 1× and centrifuged again at 10,000× *g* rpm for 10 min at room temperature. The supernatant was removed, and the pellet resuspended in 500 µL of coloration mix (1 µL of each Syto 9 and propidium iodide (LIVE/DEAD^®^ BacLight^TM^ Bacterial Viability Kit, for microscopy, Molecular probes) per 1 mL of PBS) and the solution was placed in a dark place at room temperature for 30 min. The suspension was then centrifuged at 10,000 rpm for 10 min and the supernatant removed. The pellet was resuspended in 20 µL of PBS 1× and 10 µL of the suspension placed in a glass slide. The samples were observed by fluorescence microscopy, using G-2A and FITC filters through an amplification of 1000×.

### 4.11. Gram Staining

In order to assess the *E. coli* ATCC 25922 cell morphology during the LIVE/DEAD assay, a Gram stain procedure was performed at the initial and final timepoints (0 h and 24 h). Briefly, 10 µL of each bacterial suspension was air dried and heat fixed on a slide. Then, the suspension was stained with crystal violet solution (Química Clínica Aplicada, Spain) for 1 min, followed by iodine solution (Química Clínica Aplicada) for 1 min and a mixture of acetone and alcohol (3:7) (Química Clínica Aplicada). Finally, the sample was stained by safranin (Química Clínica Aplicada) for 1 min.

### 4.12. Statistical Analyses

Statistical analyses were performed using Microsoft Excel 2019 and GraphPad Prism 6. The results presented are the mean of three independent experiments conducted ± the standard error of the mean. Unpaired *t*-test was used to measure the difference between two means in the membrane permeability assays (Figure 3) and in the biofilm formation assays (Figure 4). The two means are obtained from the experiments without dipeptide and in presence of dipeptide at the given concentration. Differences at the 5% confidence level were considered significant.

## 5. Conclusions

FF and LS have potential as antimicrobial for three clinically important bacterial species. This work point to a moderate effect in causing bacterial cell death and a strong effect in inhibiting bacterial growth and biofilm formation. Tests with four dipeptides that form channels with different diameters show that the ability to transport water molecules seems important to gain antibacterial activity and thus FF and LS may indeed form pores in the bacterial membranes. Here, we showed for the first time that FF antibacterial activity is not unique and is shared by at least another dipeptide that produces nanotubular structures (LS).

Interestingly, FF at 1.5 mg/mL, unlike LS, inhibits biofilm formation of *S. aureus* significantly and of *E. coli* moderately. Moreover, FF exerts a strong inhibition of bacteria’s growth. Our results with FF and LS assemblies, taken together, point to another mechanism, distinct from the formation of membrane pores since the two peptides produce a similar effect in planktonic (liquid, free-floating) bacteria, to be responsible for the antibiofilm activity. FF forms amyloid-like structures that reveal strong adhesion properties. Possibly FF assemblies cover surfaces, isolate and disperse bacteria preventing the formation of biofilms and inhibiting bacteria growth. Interestingly, FF is a key motif of the amyloid β-peptide and entrapment of isolated microbes by amyloid β-peptide oligomers, inhibiting pathogen adhesion, was already proposed as a mechanism of protection against microbial infection.

## Figures and Tables

**Table 1:** Antibacterial activity of supramolecular dipeptides against reference strains. MIC in mg/mL.

	*E. coli* ATCC 25922		*P.aeruginosa* ATCC 27853		*E. faecalis* ATCC 29212		*S. aureus* ATCC 29213	
	MIC	MBC	MIC	MBC	MIC	MBC	MIC	MBC
FF	1.5	>1.5	>1.5	>1.5	0.4/0.75	>1.5	1.5	1.5
FF·I_2_	>1.5	>1.5	>1.5	>1.5	0.4/0.75	1.5	1.5	1.5
LS	>1.5	>1.5	>1.5	>1.5	0.4/0.75	>1.5	1.5	1.5
LS·I_2_	1.5	1.5	>1.5	>1.5	0.4/0.75	>1.5	0.75/1.5	>1.5
IV	>1.5	>1.5	>1.5	>1.5	>1.5	>1.5	>1.5	>1.5
IV·I_2_	>1.5	>1.5	>1.5	>1.5	>1.5	>1.5	>1.5	>1.5
VI	>1.5	>1.5	>1.5	>1.5	>1.5	>1.5	>1.5	>1.5
VI·I_2_	>1.5	>1.5	>1.5	>1.5	>1.5	>1.5	>1.5	>1.5

MIC: minimal inhibitory concentration; MBC: Minimal bactericidal concentration.

**Table 2:** Fractional inhibitory concentration (FIC) index results obtained with FF or LS and kanamycin combinations by checkerboard method.

Bacterial Strain	FF + Kanamycin		LS + Kanamycin	
	ΣFIC	Activity	ΣFIC	Activity
*E. faecalis* ATCC 29212	0.26	synergism	0.26	synergism

## Data Availability

Not applicable.
